# Effects of prolonging the interval from progestin removal to prostaglandin F_2α_ injection from 16 to 17 d in a long-term estrus synchronization protocol in beef heifers^1^

**DOI:** 10.1093/tas/txab062

**Published:** 2021-04-07

**Authors:** Nicola Oosthuizen, Gabriela D Melo, George E Seidel, R Lawton Stewart, Larry Rowden, Graham C Lamb, Pedro L P Fontes

**Affiliations:** 1 Department of Animal Science, Texas A&M University, College Station, TX 77843-2471, USA; 2 Department of Biomedical Sciences, Animal Reproduction and Biotechnology Laboratory, Colorado State University, Fort Collins, CO 80523, USA; 3 Department of Animal and Dairy Science, University of Georgia, Athens, GA 30602, USA; 4 ABS Global, Broken Bow, NE 68822, USA

**Keywords:** beef heifers, estrus synchronization, fixed-time artificial insemination

## Abstract

To determine effects of delaying the injection of prostaglandin F_2α_ (PGF) and fixed-time artificial insemination (TAI) in the 14-d CIDR-PG protocol, 1,049 Angus heifers at six locations were enrolled in a completely randomized design. Within location heifers were randomly assigned to one of two treatment groups: 1) PG16 (*n* = 518), heifers received a controlled internal drug release (CIDR) insert on d 0 for 14 d, a 25-mg injection of PGF 16 d after CIDR removal (d 30), and a 100-µg injection of gonadotropin-releasing hormone concurrent with TAI 66 ± 2 h later; or 2) PG17 (*n* = 531), heifers were treated the same as PG16, however, PGF was administered 17 d after CIDR removal (d 31), and heifers were TAI 66 ± 2 h later. Estrus detection patches were applied to a subset (*n* = 482) of heifers at the time of PGF administration and were examined for activation at TAI. Dominant follicle diameter was determined via transrectal ultrasonography at PGF administration and TAI in a subset of heifers (*n* = 116). Transrectal ultrasonography was performed to determine pregnancy rates to TAI (PR/AI) between 30 and 45 d after TAI. Estrus expression prior to TAI differed by treatment where PG17 heifers had greater (*P* < 0.01) expression of estrus than PG16 heifers (57.8 ± 6.1% vs. 43.4 ± 6.1%, respectively). Nevertheless, dominant follicle diameters at PGF and at TAI were similar (*P* ≥ 0.59) between PG16 and PG17 heifers. In addition, PR/AI did not differ (*P* = 0.29) between PG16 and PG17 treatments (50.5 ± 3.2% vs. 45.7 ± 3.1%, respectively). Results of this experiment indicate that delaying the injection of PGF and TAI in the 14-d CIDR-PG protocol increased estrus expression prior to TAI yet did not improve fertility in beef heifers.

## INTRODUCTION

Estrus synchronization has been available as a reproductive management tool for over four decades and may be used to increase the proportion of beef females becoming pregnant earlier in the breeding season, and as a result, alters the calving distribution and improves calf crop uniformity (Rodgers et al., 2012). Heifers that calve earlier in the calving season have greater longevity in the herd and produce more kilograms of weaned calves compared with heifers that calve later in the season (Cushman et al., 2013). Therefore, estrus synchronization of replacement beef heifers has the ability to impact lifetime productivity by increasing the proportion of heifers that calve early in the calving season. In addition, current estrus synchronization protocols combined with fixed-time artificial insemination (TAI) have achieved pregnancy rates to TAI (PR/AI) similar to those that make use of estrus detection; therefore, estrus detection and its associated labor can be reduced or removed completely (Lamb et al., 2006; Larson et al., 2006).

Numerous short- and long-term estrus synchronization protocols are available for use in beef females, of which two long-term protocols are currently recommended for use in replacement beef heifers, namely the MGA-PG and 14-d CIDR-PG protocols (Beef Reproductive Task Force, 2021). During the 14-d CIDR-PG protocol, heifers receive a controlled internal drug release (CIDR) insert for 14 d followed by an injection of prostaglandin F_2α_ (PGF) 16 d later, and gonadotropin-releasing hormone (GnRH) administration in combination with TAI 66 h after PGF. Fertility after use of these long-term protocols is generally satisfactory; however, opportunities for improved fertility have not fully been explored.

After CIDR removal in the 14-d CIDR-PG protocol, the majority of heifers return to estrus within 48 h (~45%), but a proportion of heifers (~23%) also return to estrus between 48 and 96 h (Tauck et al., 2007; Mallory et al., 2010). Because of this variation in estrus expression after CIDR removal, intrinsic variation in follicular dynamics among heifers with multiple follicular waves, and the lack of GnRH administration to aid in controlling the ovulatory wave, the ovulatory wave might not be synchronized to the same extent as in short-term protocols where GnRH is administered within 7 d of PGF. As a result of reduced synchrony of the ovulatory wave, timing of estrus and ovulation before TAI may be suboptimal. However, additional animal handling events are undesirable in TAI protocols for beef cattle; therefore, determining the optimal timing of the current hormone regimen is likely the better alternative to increase fertility in long-term protocols than addition of hormone treatments. Research into optimal timing of PGF administration in the MGA-PG protocol demonstrated that delaying the injection of PGF from d 17 to 19 increased estrus synchrony and shortened the interval from PGF administration to estrus expression in beef heifers (Deutscher, 2000; Lamb et al., 2000). These results may be attributed to the size of the preovulatory follicle at the beginning of proestrus, as heifers that undergo luteolysis later are likely to have larger follicles at the beginning of proestrus, and thus, a reduced interval to estrus (Sirois and Fortune, 1988). Nevertheless, to our knowledge, research in delaying the injection of PGF in the 14-d CIDR-PG protocol has not previously been performed. Therefore, the objective of this experiment was to determine whether delaying PGF administration and TAI during the 14-d CIDR-PG protocol enhances fertility in beef heifers. We hypothesized that delaying the administration of PGF and TAI might result in more preovulatory follicles of a greater diameter at the time of PGF-induced luteolysis, increased estrus expression prior to TAI, and increased PR/AI when compared with heifers in the control treatment.

## MATERIALS AND METHODS

All heifers were handled in accordance with procedures approved by the University of Georgia’s Animal Care and Use Committee.

### Animals and Treatments

**Figure 1.**
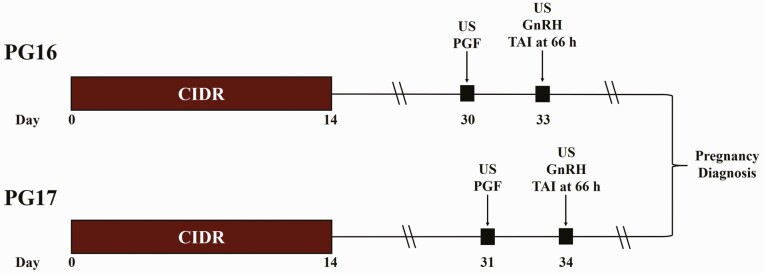
Schematic of treatments. US = transrectal ultrasonography. Breeding indicator patches were applied to a subset of heifers (*n* = 482) at PGF administration and evaluated for activation at their respective time of TAI. Pregnancy diagnosis was performed between 30 and 45 d after TAI.

A total of 1,049 *Bos taurus* beef heifers from six locations across four states (Colorado, Georgia, Nebraska, and Texas) were enrolled in this study (Table 1). Within location, heifers were randomly assigned to one of two treatment groups (Fig. 1): 1) PG16 (*n* = 518), heifers were exposed to the 14-d CIDR-PG protocol wherein they received a CIDR insert (EAZI-BREED CIDR; 1.38 g P4; Zoetis Animal Health, Parsippany, NJ) on d 0 for 14 d, a 25-mg injection of PGF (im; Lutalyse HighCon; dinoprost tromethamine; Zoetis Animal Health) 16 d after CIDR removal (d 30), and a 100-µg injection of GnRH (im; Factrel; gonadorelin hydrochloride; Zoetis Animal Health) administered at TAI 66 ± 2 h later; or 2) PG17 (*n* = 531), heifers were treated the same as PG16; however, PGF was administered 17 d after CIDR removal (d 31), and heifers were TAI 66 ± 2 h later. Heifers at three locations (*n* = 482) were fitted with breeding indicator patches (Estrotect; Rockway Inc., Spring Valley, WI) at PGF administration, which were evaluated for activation at TAI to determine expression of estrus. Breeding indicator patches were considered activated when at least 50% of the rub-off coating was removed from the patch or when the patch was missing. On d 14 heifer body weight (BW) was recorded at four locations (*n* = 523) and body condition score (BCS) was determined at two locations (*n* = 237) as previously reported (Wagner et al., 1988). Each location provided their own AI technician(s), and conventional semen. Clean-up bulls were introduced no less than 8 d after TAI at each location.

**Table 1.** Descriptive data by location^*a*^

Location	No. heifers	Breed	Mean BW, kg^*b*^	Mean BCS^*c*^
1	376	Angus	—	—
2	90	Angus	385.0 ± 3.65	—
3	96	Angus	368.6 ± 3.56	5.08 ± 0.05
4	151	Angus	377.6 ± 2.85	—
5	195	Angus	355.0 ± 2.37	—
6	141	Angus	—	5.55 ± 0.04

^*a*^Six locations across four states.

^*b*^Body weight (BW) was recorded at CIDR removal.

^*c*^Body condition score (BCS) was recorded at CIDR removal on a scale of 1 (emaciated) to 9 (obese).

### Ultrasonography

Transrectal ultrasonography (Ibex EVO II portable ultrasound, 5.0-MHz linear multifrequency transducer, Ibex, E.I. Medical Imaging, Loveland, CO) was performed at PGF administration and TAI in a subset of heifers (*n* = 116) at locations 2 and 5 to determine dominant follicle diameter. The length and width of the largest follicle on each ovary were measured using electronic calipers, and the average of the two measurements was used to reflect the diameter of the follicle. All follicles with diameters of ≥6 mm at PGF and ≥8 mm at TAI were recorded. Heifers with a dominant follicle at PGF and with no dominant follicle at TAI were considered to have ovulated prior to TAI. At each location, transrectal ultrasonography was performed between 30 and 45 d after TAI to determine PR/AI. Three heifers were unable to undergo pregnancy diagnosis.

### Statistical Analyses

All data were analyzed as a completely randomized design using the SAS statistical package (version 9.4; SAS/STAT, SAS Inst. Inc., Cary, NC). Heifer was the experimental unit in all analyses. The GLIMMIX procedure of SAS was used to analyze the binary response variables (estrus expression, PR/AI), as well as the continuous response (follicle diameter at PGF and TAI, follicle growth, and follicle growth rate) and descriptive variables (BW and BCS). To confirm that no location by treatment interaction existed, PR/AI were evaluated using treatment, location, and their respective interaction as fixed effects. No treatment by location interaction was found. Therefore, the final model for PR/AI included the fixed effects of treatment, estrus expression, and their respective interaction as well as the random effect of location. All other models for binary and continuous variables included the fixed effect of treatment and the random effect of location. Denominator degrees of freedom were adjusted using the Satterthwaite adjustment for the tests of fixed effects. Insemination technician and sire were not included in any of the models because they were equally distributed among treatments within each location. Statistical significance was declared at *P* ≤ 0.05, with 0.05 < *P* ≤ 0.10 considered a tendency. Least squares means ± SEM are reported.

## RESULTS

Descriptive variables are presented in Table 1 by location. Body weight at four locations (368.97 ± 34.5 kg) and BCS at two locations (5.36 ± 0.6) did not differ (*P* ≥ 0.11) between treatments.

A summary of ovarian response variables is presented in Table 2. Dominant follicle diameter at the time of PGF injection did not differ (*P* = 0.59) between PG16 and PG17 heifers. In addition, no difference (*P* = 0.70) in dominant follicle diameter at TAI was determined between treatments. Follicular growth between PGF and TAI (*P* = 0.45), and follicular growth rate (*P* = 0.45) did not differ between treatments. Furthermore, the percentage of heifers that ovulated between PGF and TAI did not differ (*P* = 0.33) between PG16 and PG17 treatments, and in total, 8.6% of heifers ovulated prior to TAI.

**Table 2.** Ovarian response variables measured in a subset (*n* = 116) of heifers

	Treatment^*a*^		
Item	PG16	PG17	*P* value
No. of heifers	52	64	
Follicle diameter at PGF, mm	9.29 ± 0.2	9.46 ± 0.2	0.59
Follicle diameter at TAI, mm	12.30 ± 0.2	12.18 ± 0.2	0.70
Follicle growth, mm^*b*^	3.20 ± 0.2	3.00 ± 0.2	0.45
Follicle growth rate, mm/d^*c*^	1.16 ± 0.1	1.09 ± 0.1	0.45
Ovulation rate before TAI, % (*n*)	5.8 (3/52)	10.9 (7/64)	0.33

^*a*^PG16 = heifers synchronized with the 14-d CIDR-PG protocol and received the injection of PGF 16 d after CIDR removal; PG17 = heifers synchronized with the 14-d CIDR-PG protocol but received the injection of PGF 17 d after CIDR removal and were also fixed-TAI a day later. Least square means ± SEM are reported.

^*b*^The difference between follicular diameter at PGF (d 30/31) and TAI (d 33/34).

^*c*^Follicular growth rate was calculated as the difference between follicle diameter at PGF and TAI divided by the number of d (2.75) between these two measurements.

**Figure 2.**
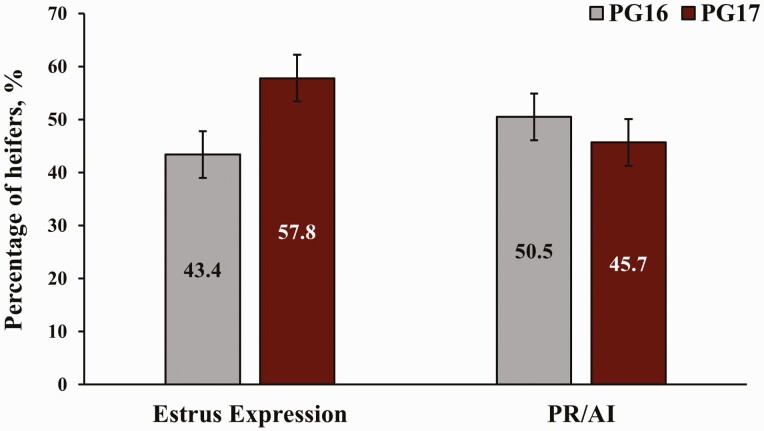
Estrus expression and PR to fixed-TAI (PR/AI) by treatment. PG16 = heifers were synchronized with the 14-d CIDR-PG protocol and received the injection of PGF 16 d after CIDR removal; PG17 = heifers were synchronized with the 14-d CIDR-PG protocol and received the injection of PGF 17 d after CIDR removal and were also TAI a day later. Breeding indicator patches were applied to all heifers at the time of the PGF injection and were evaluated for activation at their respective times of TAI. Pregnancy rates were determined via transrectal ultrasonography between 30 and 45 d after TAI. ^a,b^Within variables, bars with different superscripts differ (*P* < 0.05).

The percentage of heifers expressing estrus, as determined by activated breeding indicator patches, is presented in Fig. 2. In total, 48.9% of heifers expressed estrus. A greater percentage (*P* < 0.01) of PG17 heifers expressed estrus between injection of PGF and TAI compared with PG16 heifers.

**Figure 3.**
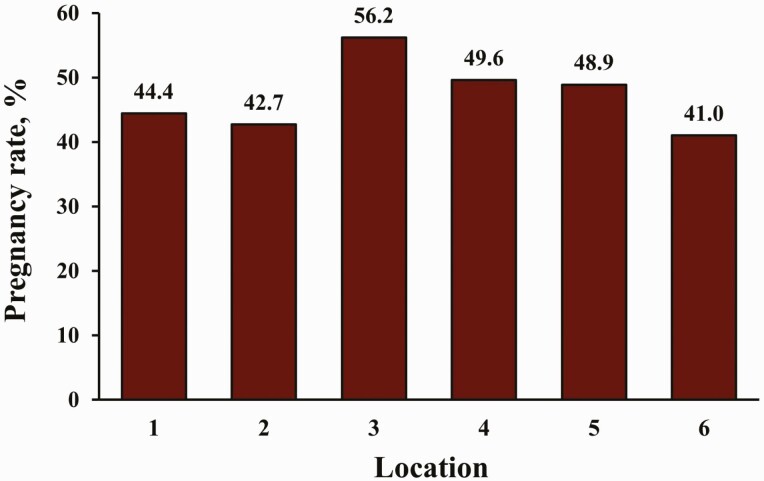
Pregnancy rates to fixed-TAI by location. Pregnancy rates were determined via transrectal ultrasonography between 30 and 45 d after TAI. No effect of location (*P* = 0.18) was determined.

**Figure 4.**
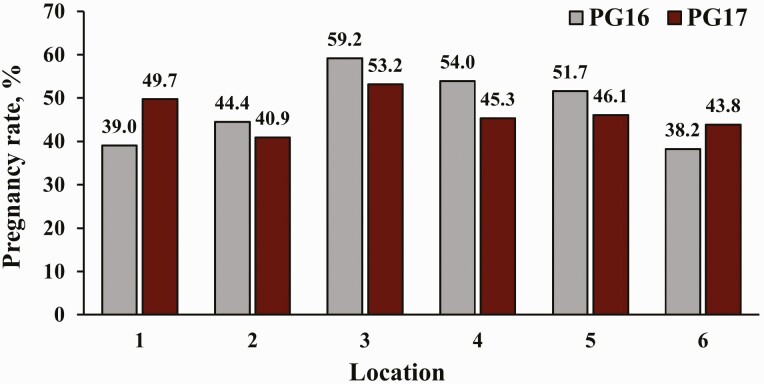
Pregnancy rates to fixed-TAI by treatment and location. PG16 = heifers were synchronized with the 14-d CIDR-PG protocol and received the injection of PGF 16 d after CIDR removal; PG17 = heifers were synchronized with the 14-d CIDR-PG protocol and received the injection of PGF 17 d after CIDR removal and were also TAI a day later. Pregnancy rates were determined via transrectal ultrasonography between 30 and 45 d after TAI. No location by treatment interaction (*P* = 0.24) was determined.

Pregnancy rates to TAI are also presented in Fig. 2. Heifers that expressed estrus had greater PR/AI (*P* < 0.01) than those that did not express estrus (61.6% vs. 34.6%, respectively). Nevertheless, PR/AI did not differ (*P* = 0.29) between PG16 and PG17 heifers, and there was no treatment by estrus expression interaction (*P* = 0.13) for PR/AI. Pregnancy rates to TAI by location are presented in Fig. 3. No effects of location (*P* = 0.18) or treatment by location interaction (*P* = 0.24) were determined for PR/AI (Fig. 4). In total, 46.5% of heifers became pregnant to TAI; PR/AI ranged from 41.0% to 56.2% among locations.

## DISCUSSION

The goal of this experiment was to determine effects of delaying the injection of PGF and TAI on estrus expression and PR/AI in replacement beef heifers. We hypothesized that heifers in the delayed group would have larger follicles at PGF, greater expression of estrus prior to TAI, and thus greater PR/AI than heifers in the control treatment. Our results indicate that although estrus expression was greater in delayed heifers, follicle size at PGF and PR/AI did not differ between treatments.

Prolonged use of a progestin is able to induce cyclicity in prepubertal heifers (Gonzalez-Padilla et al., 1975); however, fertilization of the first oocyte released after progestin removal results in reduced fertility due to formation of persistent follicles (Trimberger and Hansel, 1955; Mihm et al., 1994). Therefore, protocols such as the MGA-PG and 14-d CIDR-PG were designed to avoid AI at the first ovulation following progestin removal. Based on transrectal ultrasonography, heifers typically have three follicular waves during their 21-d estrous cycle, starting on d 2, 9, and 16 (Sirois and Fortune, 1988), and the majority of cattle will respond to an injection of PGF between d 5 and 16 (Rowson et al., 1972). Therefore, a period of 17 d between MGA removal and PGF administration was initially established to induce luteolysis during diestrus and to synchronize estrus in a majority of heifers (Brown et al., 1988). However, subsequent research demonstrated that when the injection of PGF was delayed from d 17 to 19, heifers had a greater synchronized estrus response and shorter intervals to estrus after PGF administration (Deutscher, 2000; Lamb et al., 2000). Furthermore, a greater proportion of heifers were AI within 72 h (Lamb et al., 2000) and PRs after 5 d of AI were greater in the heifers that had the PGF injection delayed to 19 d after progestin cessation (Deutscher, 2000). Nevertheless, no previous research has evaluated a prolonged interval from progestin removal to PGF administration in the 14-d CIDR-PG protocol. One previous report (Tauck et al., 2007) utilized an injection of PGF 17 d after CIDR removal in heifers; however, no comparison was made to the current standard 16 d period, and a combination of estrus detection and TAI were performed after PGF administration.

Proestrus is characterized by a rapid decline in concentrations of progesterone as a result of luteal cell apoptosis and consequent corpus luteum regression induced by PGF. This decrease in progesterone results in a decreased negative feedback to the hypothalamus and allows for increases in GnRH, follicle stimulating hormone, and luteinizing hormone secretion, ultimately leading to an increase in follicle size, estradiol secretion, and the onset of behavioral estrus, which marks the end of proestrus (Rahe et al., 1980). Therefore, the length of proestrus and interval to estrus are determined by the size of the preovulatory follicle at initiation of proestrus, as smaller follicles take longer to reach a peak of estradiol secretion and result in a lengthened proestrus period (Sirois and Fortune, 1988). During the 14-d CIDR-PG protocol, approximately 54% to 75% of heifers exhibit estrus by 60 h after PGF administration and approximately 20% to 38% exhibit estrus between 60 and 96 h (Leitman et al., 2008, 2009; Mallory et al., 2010). These ranges in time to estrus may be a result of variation in follicular growth after CIDR removal and lack of synchrony of the ovulatory wave. In the present experiment, although follicle size at PGF administration did not differ between treatments, a greater percentage of heifers in the delayed PGF treatment expressed estrus. Thus, it is plausible that greater estrus synchrony was achieved in PG17 heifers by delaying the injection of PGF, which is similar to the reports of a greater synchronized estrus response after delaying PGF administration in the MGA-PG protocol (Deutscher, 2000; Lamb et al., 2000).

Pregnancy rates to TAI are greater in cows and heifers that express estrus before TAI, where PR/AI between 11% and 43% greater have been reported (Perry et al., 2007; Richardson et al., 2016; Oosthuizen et al., 2020, 2021). Therefore, it was expected that PR/AI in the present experiment would be greater in heifers that expressed estrus when compared with those that did not express estrus. Nevertheless, although estrus expression was greater in PG17 heifers, PR/AI did not differ between treatments. This lack of difference in PR/AI may be due to the magnitude of the increase in estrus expression, which may not have been great enough to increase PR/AI. It is also conceivable that more heifers expressed estrus sooner after PGF administration, which may have altered the optimal timing of TAI to occur sooner after PGF and may have reduced PR/AI in that proportion of heifers, resulting in no difference in PR/AI between treatments. Lastly, the number of animals in this study may not have been great enough to eliminate the possibility of a difference between PR/AI and may have resulted in a type II statistical error; therefore, these results should be interpreted with caution. Future research is required to corroborate the results of this study and should evaluate the distribution of estrus after delayed PGF administration in order to determine the optimal timing of TAI, as there may be opportunities to increase PR/AI.

In conclusion, estrus expression was greater in PG17 heifers when compared with PG16 heifers; however, PR/AI did not differ between treatments. Therefore, delaying the administration of PGF as well as TAI in the 14-d CIDR-PG protocol does not improve fertility in beef heifers, yet may potentially provide some flexibility in scheduling for beef cattle producers.

*Conflict of interest statement*. None declared.
